# Microbiome and metabolome integrated analysis: exploring potential diagnostic approaches for Parkinson’s disease using tongue coating samples

**DOI:** 10.3389/fmicb.2025.1621468

**Published:** 2025-07-29

**Authors:** Runjuan Yang, Mengqi Jia, Ying Xu, Zhenghua Wu, Dongying Wu, Yaxing Gui

**Affiliations:** 1 Department of Clinical Pharmacy, Shanghai General Hospital, Shanghai Jiao Tong University School of Medicine, Shanghai, China; 2 Department of Neurology, Shanghai General Hospital, Shanghai Jiao Tong University School of Medicine, Shanghai, China; 3 School of Medicine, Shanghai University, Shanghai, China

**Keywords:** microbiomics, 16S rRNA, metabolomics, UPLC-Q/TOF-MS, Parkinson’s disease, tongue coating

## Abstract

Parkinson’s disease (PD) is a prevalent neurodegenerative disorder with complex pathogenesis and limited treatment options. The current reliance on clinical evaluation for diagnosis, due to the absence of reliable non-invasive methods, presents significant challenges. Traditional diagnostic approaches, including cerebrospinal fluid or blood sampling, are invasive, pose risks of infection, are costly, and often require complex procedures. Tongue coating sampling presents a non-invasive, cost-effective, and repeatable alternative, indicating that it could be a valuable tool for early detection and monitoring of PD, warranting further investigation. This study explores the feasibility of using tongue coating samples as a diagnostic tool for PD through microbiome analysis, with metabolomics data providing additional context and validation via machine learning models. A cohort of 36 PD patients and 31 controls was recruited. 16S rRNA sequencing was used for microbiome analysis, revealing significant alterations in the relative abundances of various microbial taxa, including Firmicutes, Bacteroidetes, and Actinobacteria. Concurrent metabolomics analysis using UPLC-Q/TOF-MS revealed a decrease in palmitoylethanolamide (PEA) levels in Parkinson’s disease (PD) patients, and also showed reduced carnitine levels specifically in the severe Hoehn-Yahr (H-Y) stage and mild cognitive impairment (MCI) subgroups. These findings provide preliminary evidence suggesting a potential link between specific microbial alterations and PD progression, which may warrant further investigation. Additionally, the analysis indicates a correlation between certain microbial and metabolomic changes and the advancement of PD. Our results also suggest that tongue coating may serve as a potential non-invasive tool for PD diagnosis, with a particular emphasis on the combined role of the microbiome and metabolome in the pathogenesis of the disease.

## 1 Introduction

Parkinson’s disease (PD) is a neurodegenerative disease characterized by resting tremors, slow movement, muscle rigidity, as well as some non-motor symptoms and secondary motor symptoms (Tolosa et al., 2006; Jankovic, 2008). Numerous environmental and genetic factors influence the risk of PD, with different factors predominating in distinct patients. These factors converge on specific pathways, including mitochondrial dysfunction, oxidative stress, protein aggregation, impaired autophagy, and neuroinflammation (Simon et al., 2020). It is the second most prevalent neurodegenerative disease (Ben-Shlomo et al., 2024). In high-income countries, the median age-standardized annual incidence rate of PD is approximately 14 cases per 100,000 individuals (Ascherio and Schwarzschild, 2016). Currently, the diagnosis of PD is based on clinical criteria, with the standard definition encompassing bradykinesia accompanied by resting tremor, rigidity, or both(Bloem et al., 2021). The current treatment for PD primarily involves levodopa to alleviate motor symptoms, supplemented by dopamine agonists or B-type monoamine oxidase inhibitors based on individual patient profiles (Foltynie et al., 2024). For all PD patients, current therapies are symptomatic, focusing on improving motor and non-motor signs and symptoms, with no medications demonstrating definitive evidence of disease-modifying effects (Armstrong and Okun, 2020; Vijiaratnam et al., 2021). However, PD may have a prodromal phase during which precise and early diagnosis based on clinical presentation remains challenging (Bloem et al., 2021). In addition, clinical diagnosis of PD typically achieves an accuracy of only 80–90% when compared to pathological confirmation (Ascherio and Schwarzschild, 2016). This urgency has driven significant interest in the study of biomarkers for PD, highlighting the critical need for more convenient, accurate, and early diagnostic methods. Currently, the primary sample sources for clinical research on biomarkers for PD are blood and cerebrospinal fluid (CSF) (Siderowf et al., 2010; Aasly et al., 2012; Abbasi et al., 2018; Ng et al., 2020; Aamodt et al., 2021; Mao et al., 2023). However, both blood and cerebrospinal fluid collection are invasive procedures, which can impose substantial psychological stress and financial burden on patients.

During the patients’ clinical visits, we observed that the tongue coating (TC)of PD patients exhibited a notable thick and greasy appearance, with some presenting abnormal white or yellow discoloration. This piqued our interest. TC refers to the layer present on the dorsal surface of the tongue, which serves as a critical observation target in the “visual diagnosis” of traditional Chinese medicine (TCM). It is primarily composed of a complex mixture of microorganisms (bacteria), keratinized epithelial cells, saliva, blood metabolites, and food residues (Danser et al., 2003; Van Tornout et al., 2013; Seerangaiyan et al., 2018). The appearance of TC is typically described based on its color and texture. Coating colors are generally classified into four main categories: white, yellow, gray, and black. Texture, on the other hand, encompasses various types, including thin, thick, smooth, dry, greasy, rotten, peeled, and the presence or absence of TC roots. While the study of TC has been extensively explored within the framework of traditional Chinese medicine, recent research has increasingly focused on advanced techniques such as image acquisition, digital processing, and computer-aided analysis (Kim et al., 2013; Segawa et al., 2021; Yuan et al., 2023). Research has suggested that tongue images provide a stable and reliable method for diagnosing gastric cancer, offering significant advantages over traditional blood biomarkers (Yuan et al., 2023). Research on TC is rapidly advancing, with its applications gradually extending beyond oral (Zhang et al., 2024) and gastrointestinal diseases (Cui et al., 2019; Mu et al., 2019; Guo et al., 2022) to encompass a broader spectrum of health conditions (Kapila, 2021; Pathak et al., 2021). Beyond image processing, the study of TC composition has gained increasing attention. TC sampling is straightforward, non-invasive, and low-risk, making it an attractive and promising material for clinical research. Emerging evidence suggests that TC holds significant potential as a non-invasive diagnostic tool for disease detection and as a means to monitor disease progression and prognosis (Ali Mohammed et al., 2021; Li et al., 2021).

Building on the aforementioned insights, we are particularly interested in identifying biomarkers within the TC that could aid in the diagnosis and prognosis of PD. The unique composition of TC presents an untapped potential for uncovering disease-specific metabolic signatures. Ultra-Performance Liquid Chromatography coupled with Quadrupole Time-of-Flight Mass Spectrometry (UPLC-Q/TOF-MS) has been extensively applied in metabolomics research, demonstrating its capability to analyze complex biological samples and elucidate metabolic alterations in various diseases (Cui et al., 2018; Shao et al., 2021; Zhao et al., 2021; Liu et al., 2022; Yang et al., 2022). 16S rRNA (16S ribosomal RNA) sequencing technology is widely used in microbiome research as it provides insights into the composition of microbial communities. It is a reliable method for high-throughput sequencing analysis (Curry et al., 2022; Hassler et al., 2022). Thus, we propose to conduct a non-targeted metabolomics study of TC samples from Parkinson’s patients using UPLC-Q/TOF-MS. At the same time, we applied 16S rRNA sequencing to study the microbiome of PD patients, aiming to identify differences in their microbial communities. By integrating untargeted metabolomics and microbiome analysis, we hope to fill the gap in research in this area. Our goal is to identify reliable biomarkers that could serve as non-invasive diagnostic tools and provide new therapeutic targets, paving the way for innovative approaches in the diagnosis and treatment of PD.

## 2 Materials and methods

### 2.1 Materials and reagents

Methanol and acetonitrile (LC/MS grade) were purchased from Thermo Fisher Scientific (China). Ultrapure water (18.2 MΩ/cm) was produced using a Milli-Q system (Millipore, Bedford, MA, United States). Formic acid (FA) and ammonium acetate (AA) were obtained from ANPEL Lab Tech. (Shanghai, China). Quant-iT PicoGreen dsDNA Assay Kit (Invitrogen, United States) TruSeq Nano DNA LT Library Prep Kit (Illumina, Inc., United States) Agilent High Sensitivity DNA Kit (Agilent Technologies, Inc., United States).

### 2.2 Sample collection and research methods

#### 2.2.1 Sample source

**Figure 1:**
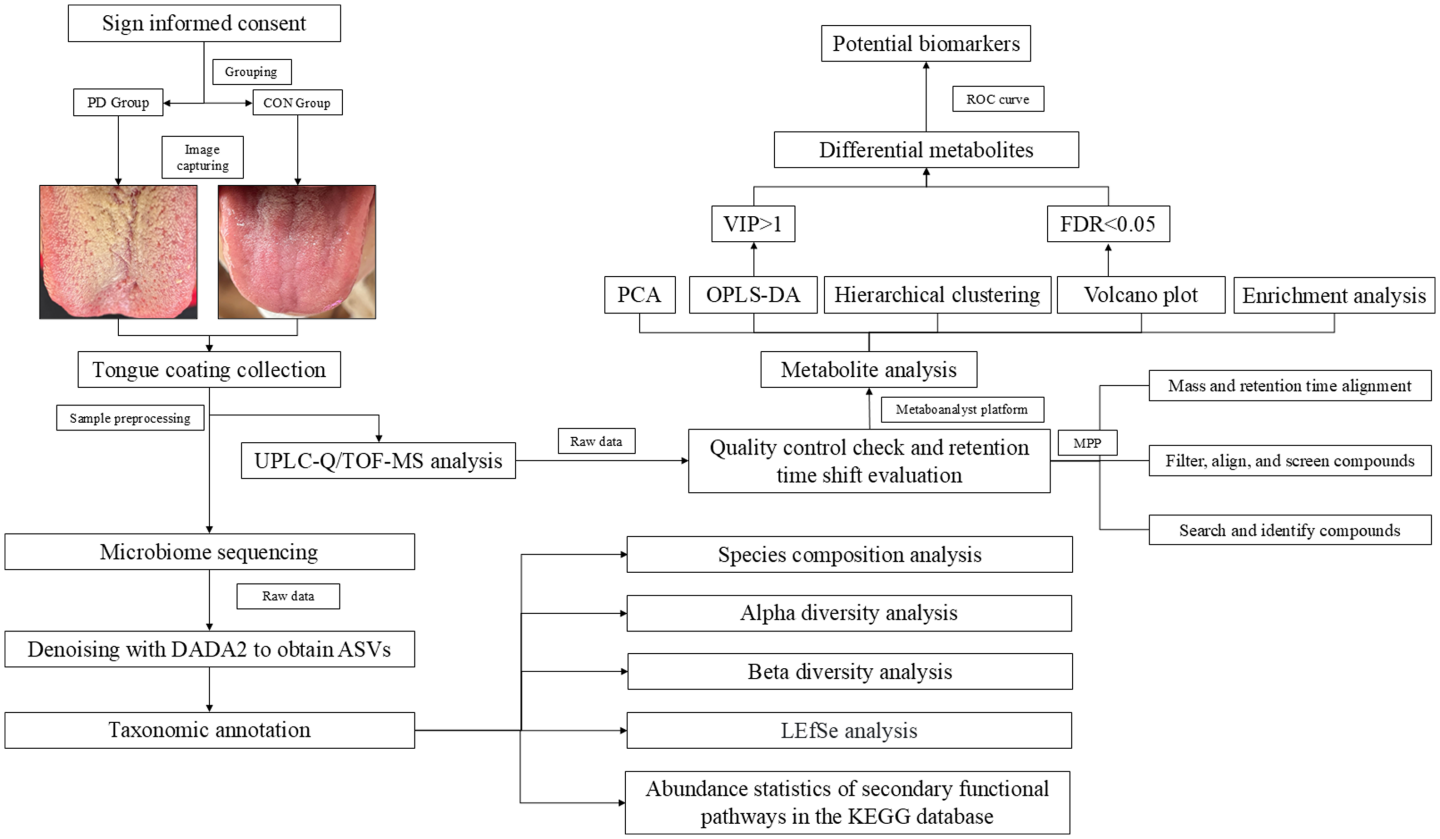
Experimental flowchart.

The PD group consisted of patients diagnosed with PD who were recruited from the Neurology Outpatient Department of Shanghai General Hospital. All participants in this group were patients undergoing follow-up visits every 4 weeks, with the total sample size based on the number of patients enrolled during one four-week cycle. The control group (CON) was composed of family members accompanying the patients, who met the inclusion criteria. All participants voluntarily took part in this study and this study was approved by the Ethics Committee of Shanghai General Hospital (Approval No. 2024HS158). The study strictly adhered to the principles outlined in the *Declaration of Helsinki* and was registered with the Chinese Clinical Trial Registry (Registration no. ChiCTR2400091883). The experimental workflow is shown in Figure 1.

#### 2.2.2 Inclusion and exclusion criteria

PD diagnostic criteria: PD group were based on the internationally recognized *MDS Clinical Diagnostic Criteria for Parkinson’s Disease* (Postuma et al., 2015).

Inclusion criteria for the PD group were as follows: (1) Fulfillment of the diagnostic criteria for PD (MDS Clinical Diagnostic Criteria). (2) Age > 55 years. (3) Willingness to participate in the study, with written informed consent provided. (4) Absence of other systemic diseases, such as malignancies, infectious diseases, or gastrointestinal disorders. (5) Samples were collected before the intake of food and Parkinson’s-related medications.

Inclusion criteria for the CON group were as follows: (1) Confirmation of no neurodegenerative diseases. (2) Age > 55 years. (3) Willingness to participate in the study, with written informed consent provided. (4) Absence of other systemic diseases, such as malignancies, infectious diseases, or gastrointestinal disorders.

Exclusion Criteria for Both Groups: (1) Use of medications affecting TC status (e.g., antibiotics, antifungal agents) within 2 weeks prior to sampling. (2) Presence of diseases that influence TC status (e.g., acute upper respiratory tract infection, oral ulcers, glossitis). (3) Coexisting major illnesses, such as liver or kidney dysfunction, severe infections, or malignancies.

#### 2.2.3 Collection and processing of TC samples

To minimize interference from residual debris, participants were instructed to avoid eating prior to TC collection. On-site, participants rinsed their mouths three times to further eliminate potential residue and saliva interference. Following rinsing, TC images were captured. TC samples were collected by scraping the dorsal surface of the tongue from left to right five times. The collected material was placed into an Eppendorf tube prefilled with 500 μL of physiological saline. The sample was then mixed thoroughly. Cell counting was performed using the Countess II Automated Cell Counter (Thermo Fisher Scientific), and the cell concentration was adjusted to ensure a uniform count of approximately 10^6 cells/mL for all samples. Finally, the samples were stored at −80°C until further analysis.

#### 2.2.4 Metabolomics processing procedure

After all samples have been collected, they are centrifuged at 1000 rpm for 4 min at 4°C. The supernatant is dried using nitrogen gas (N₂). The cell pellets are then re-suspended in 100 μL of cold 90% acetonitrile, ensuring uniform suspension of the cells. The samples undergo three freeze–thaw cycles at −80°C, each lasting 10 min, to facilitate cell disruption. The remaining 90% acetonitrile is added to bring the total volume to 200 μL for extraction, followed by vortex mixing to ensure thorough homogenization, resulting in 200 μL of acetonitrile extract. The samples are then subjected to ultrasonic treatment for 10 cycles, each lasting 30 s, to further enhance extraction. Finally, the samples are centrifuged at 15000 rpm for 10 min at 4°C to remove any residual solid material, and 150 μL of the supernatant is collected for analysis.

##### 2.2.4.1 UPLC-Q/TOF-MS instrument and conditions

Samples were analyzed using an Agilent 1,290 Infinity UPLC system (Milford, MA, United States) equipped with a Waters UPLC column (ACQUITY HSS T3, 3.0 × 150 mm, 1.8 μm). The mobile phase consisted of (A) 10 mM ammonium acetate aqueous solution with 0.1% formic acid and (B) acetonitrile. The flow rate was set at 0.3 mL/min, the column temperature was maintained at 35°C, and the injection volume was 5 μL. Gradient elution was performed as follows (Mu et al., 2019): 0–2 min, 10–50%B; 2–14.5 min, 50–95% B; 14.5–15 min, 95–10% B; 15–20 min, 10–10% B.

Mass spectrometry conditions: Mass spectrometry data were obtained using an Agilent 6,545 Q-TOF MS/MS equipped with a dual Agilent Jet Stream electrospray ionization (ESI) source operated in positive ionization mode. The parameters were set as follows: scan range of 100–1,000 m/z for MS and 50–800 m/z for MS/MS; fragmentor voltage of 175 V; gas temperature of 320°C; sheath gas temperature of 350°C; sheath gas flow rate of 11 L/min; nebulizer gas flow rate of 8 L/min; nebulizer gas pressure of 35 psig; and collision energy alternating between 10 and 30 eV.

##### 2.2.4.2 Quality control

All enrolled samples were retrieved from the −80°C freezer and allowed to reach room temperature. A 20 μL aliquot was taken from each sample and pooled to create a quality control (QC) sample, which underwent the same preprocessing and instrumental analysis as the other samples. PD group samples were injected first, followed by CON group samples. A QC sample was injected after every 10 samples to minimize errors arising from sample preprocessing and detection.

#### 2.2.5 Microbiome sample processing procedure

All enrolled samples were collected, and total DNA was extracted. DNA concentration was quantified using a Nanodrop spectrophotometer, and DNA quality was assessed by 1.2% agarose gel electrophoresis. The 16S rRNA gene V3-V4 region was amplified using the following primers: Forward primer: ACTCCTACGGGAGGCAGCA, Reverse primer: GGACTACHVGGGTWTCTAAT. The amplified products were purified using magnetic beads and then quantified via fluorescence using a Microplate Reader (BioTek, FLx800). The sequencing library was prepared using Illumina’s TruSeq Nano DNA LT Library Prep Kit. Library quality was assessed on an Agilent Bioanalyzer using the Agilent High Sensitivity DNA Kit. After the library passed quality control, it was subjected to sequencing.

#### 2.2.6 Data analysis

The measurement data are expressed as mean ± standard deviation (x̄ ± s), and categorical data are presented as frequencies and percentages. Statistical analysis of the data between groups was performed using SPSS (version 25) and R software (version 4.3.3). Age was analyzed using a t-test, while gender, the presence of hypertension, and the presence of hyperlipidemia were analyzed using chi-square (χ^2^) tests. TCS was analyzed using the Mann–Whitney U test.

The data obtained from UPLC-Q/TOF-MS were processed using MassHunter Qualitative Analysis Software (version 10.0) and Mass Profiler Professional (MPP) software (version 15.1) for the following steps: quality control checks of the raw data, retention time variation assessment, compound filtering and calibration, compound identification, and differential analysis based on mass and retention time. Identification was performed using the METLIN and HMDB databases. The processed data from MPP were then imported into the MetaboAnalyst 6.0 online platform^1^ for differential analysis, principal component analysis (PCA), partial least squares discriminant analysis (PLS-DA), sparse partial least squares discriminant analysis (sPLS-DA), orthogonal partial least squares discriminant analysis (OPLS-DA), hierarchical clustering, and enrichment analysis. PCA is an unsupervised method that aims to explain the main variations in the data by reducing its dimensionality. PLS-DA is a supervised method used to relate independent variables to categorical outcomes (i.e., class labels). OPLS-DA further enhances PLS-DA by separating orthogonal components (unrelated noise) from the model, which improves the distinction between categories. The advantage of OPLS-DA lies in its ability to improve model interpretability while reducing the influence of irrelevant data. R2Y is used to assess the model’s ability to explain the Y variable (i.e., class labels), while Q2 reflects the model’s predictive capability, determined by the cross-validation-based prediction correlation coefficient. A permutation test with 2000 iterations was performed to evaluate the model’s validity and statistical significance. In this study, compounds meeting both FDR < 0.05 and VIP > 1 were considered differential metabolites. The identified differential metabolites were submitted to the MetaboAnalyst platform for enrichment analysis. Additionally, the diagnostic capability of these differential metabolites was evaluated using ROC curve analysis. The ROC curves were generated and analyzed via the Extreme Smart Analysis platform,^2^ and the ROC data were further validated using SPSS (version 25). All the data analysis was performed using the peak area of mass spectrometry for relative quantification.

After performing an initial quality check on the raw 16S rRNA sequencing data, the samples were demultiplexed based on index and barcode information, and the barcode sequences were removed. The data were processed using QIIME2 (2019.4) software, with the DADA2 plugin for quality control, denoising, merging, and chimera removal. After denoising all libraries, the Amplicon Sequence Variants (ASVs) feature sequences and the corresponding ASV table were merged, and singleton ASVs were removed. The Greengenes database (Release 13.8) was used for taxonomic assignment of each ASV’s feature sequence in QIIME2 using the default parameters and a pre-trained Naive Bayes classifier. Taxonomic classification results at the phylum and genus levels were primarily presented. Alpha diversity indices were used to characterize the species richness, diversity, and evenness within habitats, while beta diversity indices were used to assess the differences between samples and habitats. The LEfSe (LDA Effect Size) analysis, which combines the non-parametric Kruskal-Wallis and Wilcoxon rank-sum tests with linear discriminant analysis (LDA) effect size, was used to identify robust differential species between groups. Additionally, metabolic pathway analysis was performed using the KEGG database.

TC images were primarily assessed using the TC Score (TCS) (Aimetti et al., 2015). The TCS is calculated as the product of the TC coverage area and thickness. Coverage area is scored from 0 to 3 as follows: 0 (no TC), 1 (coating covers ≤1/3 of the tongue dorsum), 2 (1/3 < coating coverage ≤ 2/3), and 3 (coating covers >2/3 of the tongue dorsum). Thickness is also scored from 0 to 3: 0 (no TC), 1 (thin coating), 2 (moderate coating with papillae not visible), and 3 (thick coating).

The Mini-Mental State Examination (MMSE) is a widely used tool to assess cognitive function. In our study, scores below 27 were classified as the mild cognitive impairment (MCI) group, while scores of 27 or above were classified as the Normal group. The Hoehn and Yahr (H-Y) scale is a clinical rating system used to assess the severity of Parkinson’s disease (PD) based on motor symptoms. The scale ranges from 1 to 5, with stages 4 and 5 categorized as the severe group, and stages 1, 1.5, 2, 2.5, and 3 categorized as the mild and moderate group. The MMSE score and H-Y stage help categorize PD patients into different stages of cognitive decline and disease progression, providing a framework for understanding the relationship between cognitive decline and disease severity.

Random Forest classification is a commonly used machine learning method for developing predictive models in various research environments (Speiser et al., 2019). It improves the accuracy of classification or regression models by constructing multiple decision trees and combining their predictions. We conducted Random Forest analysis on the metabolomics data using R (version 4.4.4). In this experiment, we first randomly selected 5 samples from the 36 Parkinson’s disease (PD) group samples as an independent validation set, ensuring that the model could later be tested. Next, we constructed a dataset using the remaining 31 PD group samples and 31 control (CON) group samples, with the data randomly split into 70% training set and 30% testing set for model training and evaluation. After the model was established, we further validated its performance by applying the model to the previously randomly selected independent validation set consisting of the 5 samples.

## 3 Results

### 3.1 Demographic characteristics

The mean age of the CON group was (71.77 ± 6.453) years, while the mean age of the PD group was (72.81 ± 7.398) years. There was no statistically significant difference in the overall age between the two groups (RD –1.031, 95% CI –4.446–2.383, *t* = −0.603, *p* = 0.584). In terms of gender, 33.3% of the PD group were female, and 54.8% of the CON group were female. No statistically significant difference was found between the two groups in terms of gender (RD 0.22, 95% CI − 0.0182–0.4483, *χ*^2^ = 3.138, *p* = 0.076). For hypertension, 58.3% of the PD group had hypertension, while 51.6% of the CON group had hypertension. There was no statistically significant difference between the two groups regarding the presence of hypertension (RD 0.07, 95% CI –0.1713–0.3057, *χ*^2^ = 0.304, *p* = 0.581). Similarly, 47.2% of the PD group had hyperlipidemia, while 48.4% of the CON group had hyperlipidemia. Again, no statistically significant difference was found between the two groups regarding the presence of hyperlipidemia (RD –0.01, 95% CI –0.2512–0.2282, *χ*^2^ = 0.009, *p* = 0.924). Therefore, neither the age, gender, hypertension, nor hyperlipidemia of participants had any impact on the experimental grouping in this study. The median TCS score for the PD group was 3 (2, 6), while the median TCS score for the CON group was 2 (2, 3). A statistically significant difference was observed in the overall TCS distribution between the two groups (Z = 2.507, *p* = 0.012) (Table 1).

**Table 1:** Demographic characteristics.

Variable	CON (*n* = 31)	PD (*n* = 36)	Test Statistic [RD (95% CI)]		*p*-value
Age (Mean±SD)	71.77 ± 6.453	72.81 ± 7.398	*t* = −0.603	–1.031(−4.446–2.383)	0.548
Gender [*n* (%)]					
Woman	17(54.8%)	12(33.3%)	χ^2^ = 3.138	0.22(−0.0182–0.4483)	0.076
Man	14(45.2%)	24(66.7%)			
TCS [M (P_25_, P_75_)]	2(2,3)	3(2,6)	Z = 2.507	1(0–4)	0.012
BMI (Mean ± SD)	22.72 ± 2.367	21.79 ± 2.985	*t* = 1.394	0.9282(−0.4015–2.2579)	0.168
Hypertension					
Yes	16(51.6%)	21(58.3%)	χ^2^ = 0.304	0.07(−0.1713–0.3057)	0.581
No	15(48.4%)	15(41.7%)			
Hyperlipidemia					
Yes	15(48.4%)	17(47.2%)	χ^2^ = 0.009	–0.01(−0.2512–0.2282)	0.924
No	16(51.6%)	19(52.8%)			

### 3.2 16S rRNA sequencing results

**Figure 2:**
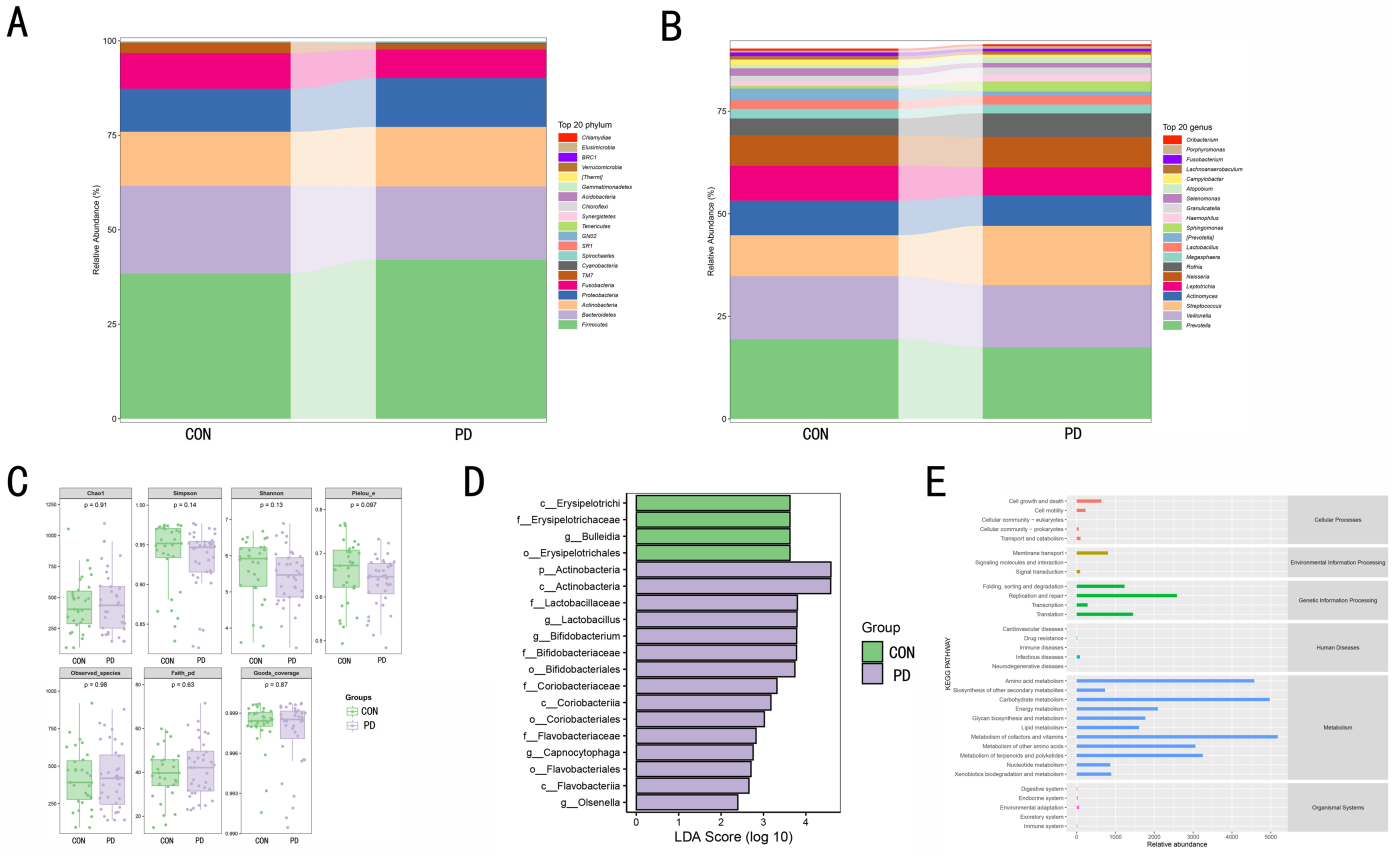
**(A)** shows the relative abundance distribution of microbial communities at the phylum level between the CON and PD groups. The relative abundance of the top 20 dominant phyla is color-coded, and the bar area represents the relative abundance of each phylum within each group. **(B)** displays the relative abundance distribution of microbial communities at the genus level between the CON and PD groups. The relative abundance of the top 20 dominant genera is color-coded, and the bar area reflects the relative proportion of each genus within each group. **(C)** presents the microbial diversity in the CON and PD groups using boxplots, including the Chao1 index, Simpson index, Shannon index, Pielou_e index, Observed_species count, and Faith_pd index. The *p*-values indicate the significance of differences in these indices between the two groups, with a significance level of *p* < 0.05 considered statistically meaningful. **(D)** shows the differential microbial taxa identified by LEfSe analysis. The length of the bars represents the LDA score, with larger values indicating more significant differences. A score greater than 2 is considered statistically significant. Green bars represent taxa enriched in the CON group, and purple bars represent taxa enriched in the PD group. **(E)** presents the relative abundance distribution of KEGG pathways predicted from functional analysis.

At the phylum level (Figure 2A), the microbial composition between the CON and PD groups showed differences. Firmicutes and Bacteroidetes were the dominant phyla in both groups, with the relative abundance of Firmicutes increased in the PD group, while Bacteroidetes showed the opposite trend. The relative abundance of Proteobacteria and Actinobacteria slightly increased in the PD group. Other phyla, such as Fusobacteria, also exhibited minor changes, but their impact was minimal. At the genus level (Figure 2B), differences were also observed among the top 20 dominant genera in the CON and PD groups. For instance, Prevotella and Veillonella, which dominated in the CON group, decreased in the PD group, while Streptococcus and Actinomyces, which had higher proportions, increased in the PD group. Other genera showed certain changes, indicating notable inter-group differences. Through *α*-diversity analysis (Figure 2C), no significant differences were observed between the CON and PD groups in terms of the Chao1, Simpson, Shannon, and Pielou_e indices (*p* > 0.05), suggesting that the overall species richness, diversity, and evenness remained relatively stable between the two groups. LEfSe analysis (Figure 2D) revealed significant differences between the CON and PD groups across multiple taxonomic levels, including phylum, class, order, family, and genus. Specifically, populations associated with Erysipelotrichaceae and Bulleidia were significantly enriched in the CON group, while taxa related to Actinobacteria, Bifidobacterium, Coriobacteriaceae, and Flavobacterium were notably enriched in the PD group. These differential microbial populations may serve as important microbial biomarkers that contribute to or reflect the pathophysiological changes in PD. In the KEGG pathway enrichment analysis (Figure 2E), we observed that pathways related to metabolism occupied a larger proportion.

### 3.3 Metabolomics data results

#### 3.3.1 Data stability and reliability

**Figure 3:**
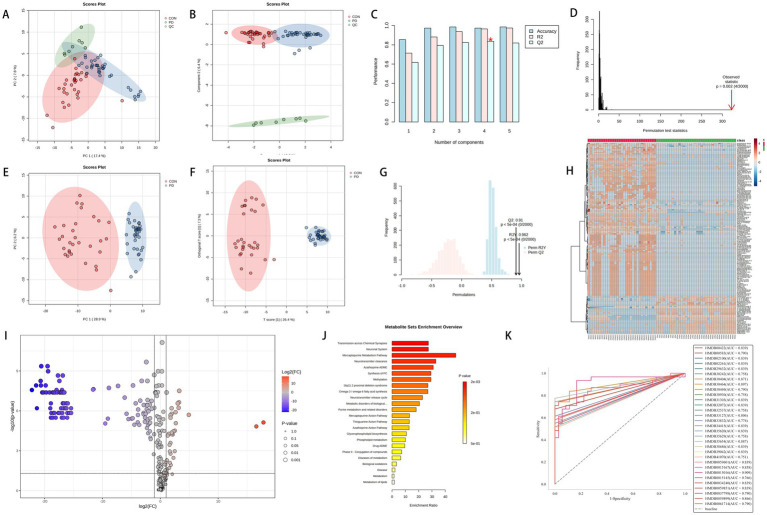
Panels **(A–D)** represent the PCA score plot, sPLS-DA score plot, bar chart of model performance metrics (Accuracy, *R*^2^, *Q*^2^) under different component numbers in PLS-DA, and the 2000-permutation test for PLS-DA, respectively, for the PD group, CON group, and QC group. Panels **(E–G)** show the PCA score plot, OPLS-DA score plot, and the 2000-permutation test for OPLS-DA, respectively, for the PD and CON groups. Panel **(H)** displays a hierarchical clustering heatmap of metabolites. Panel **(I)** shows the volcano plot of metabolites, where significantly upregulated metabolites are located in the top-right corner [log2(FC) > 1 and -Log10(*p*) > 1.3], and significantly downregulated metabolites are located in the top-left corner [log2(FC) < −1 and -Log10(*p*) > 1.3, with p-values adjusted using the Benjamini-Hochberg correction]. Panel **(J)** shows the KEGG enrichment analysis. Panel **(K)** presents the ROC analysis of 31 potential biomarkers for PD diagnosis.

In Figure 3, panels A and B show significant separation among the three groups, with the QC results indicating that the samples were stable throughout the preprocessing and analysis processes. Panels C and D suggest that the R^2^ and Q^2^ values for the first two components are both >0.7. After performing 2000 random permutations, the statistic for each permutation was calculated, yielding a *p*-value of 0.002, which indicates statistical significance. This suggests that our model has strong predictive power, high classification accuracy, and low risk of overfitting. Four components are considered the optimal choice, as this configuration balances model performance and complexity.

**Figure 4:**
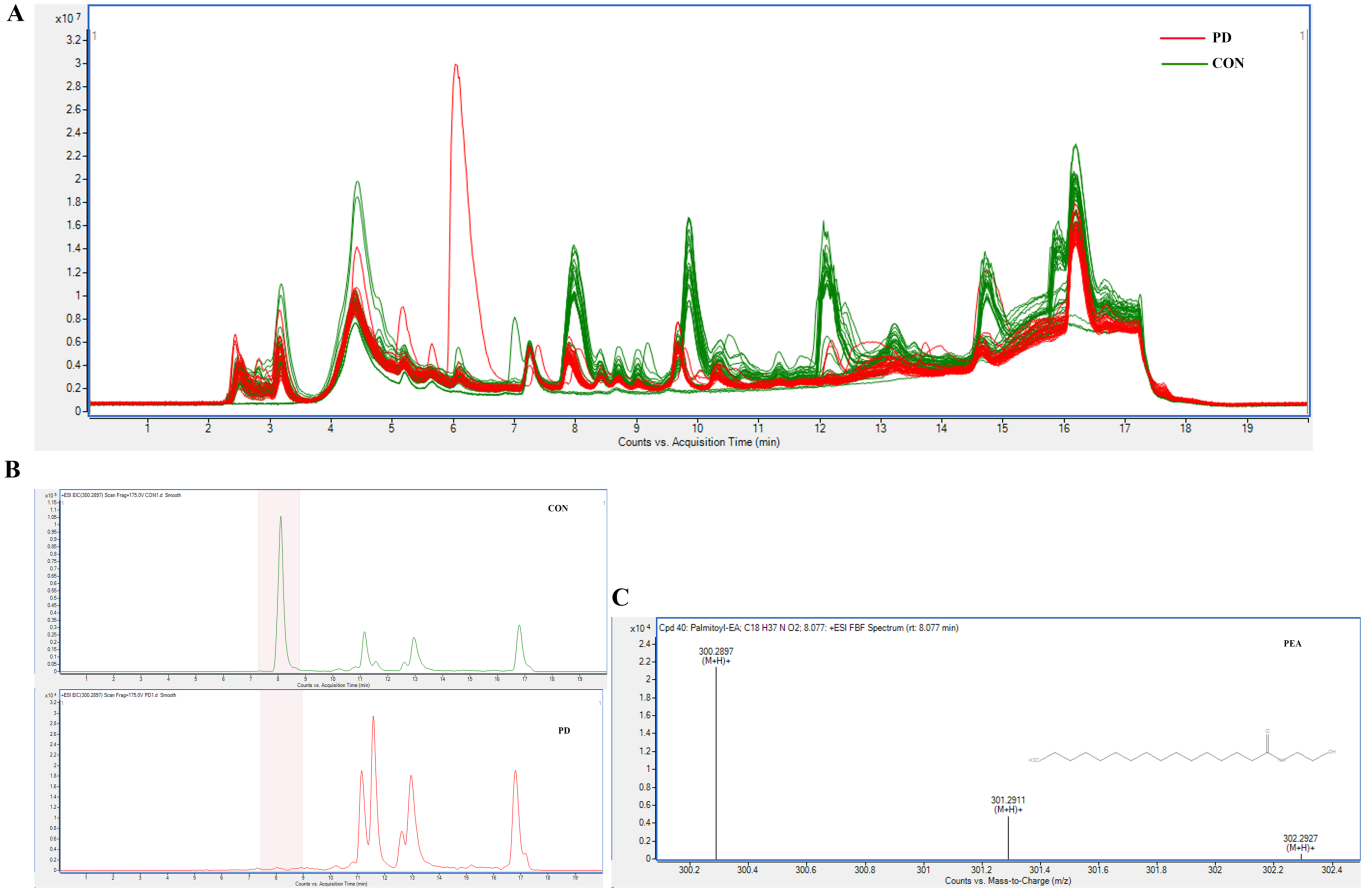
**(A)** shows the comparison of TIC chromatograms between the two groups. **(B)** illustrates the EIC chromatograms of PEA in representative samples from both groups. **(C)** presents the MS/MS identification spectrum of PEA.

The mass spectrometry TIC comparison between the PD group and the CON group is shown in Figure 4A, where a noticeable difference is observed between the two. In Figure 3, panels E and F suggest a clear separation between the two groups. Panel G shows *R*^2^Y and *Q*^2^ values > 0.8 for the model, indicating good fitting and predictive performance. The permutation test effectively prevents overfitting, further supporting the robustness and reliability of the model.

#### 3.3.2 Screening and Analysis of Potential Biomarkers

After filtering through MPP software, a total of 1,037 metabolites were identified, among which 294 were preliminarily annotated. Hierarchical clustering analysis was performed on the annotated metabolites to illustrate the similarities between different samples or metabolites. The clustering of samples was based on standardized metabolite data. Figure 3H highlights the top 150 metabolites with the highest significance in the analysis.

Figure 3I illustrates the differential expression of metabolites, with 35 upregulated and 116 downregulated metabolites identified among the 294 compounds. The upregulated metabolites may be associated with metabolic activation or promotion effects, while the downregulated metabolites may reflect suppressed metabolism or weakened activity in related pathways. Figure 3J depicts the KEGG pathway enrichment analysis of the upregulated and downregulated metabolites identified in the volcano plot. The metabolites are primarily enriched in pathways such as transmission across chemical synapses, neuronal system, mercaptopurine metabolism pathway, neurotransmitter clearance, azathioprine ADME, and methylation.

**Figure 5:**
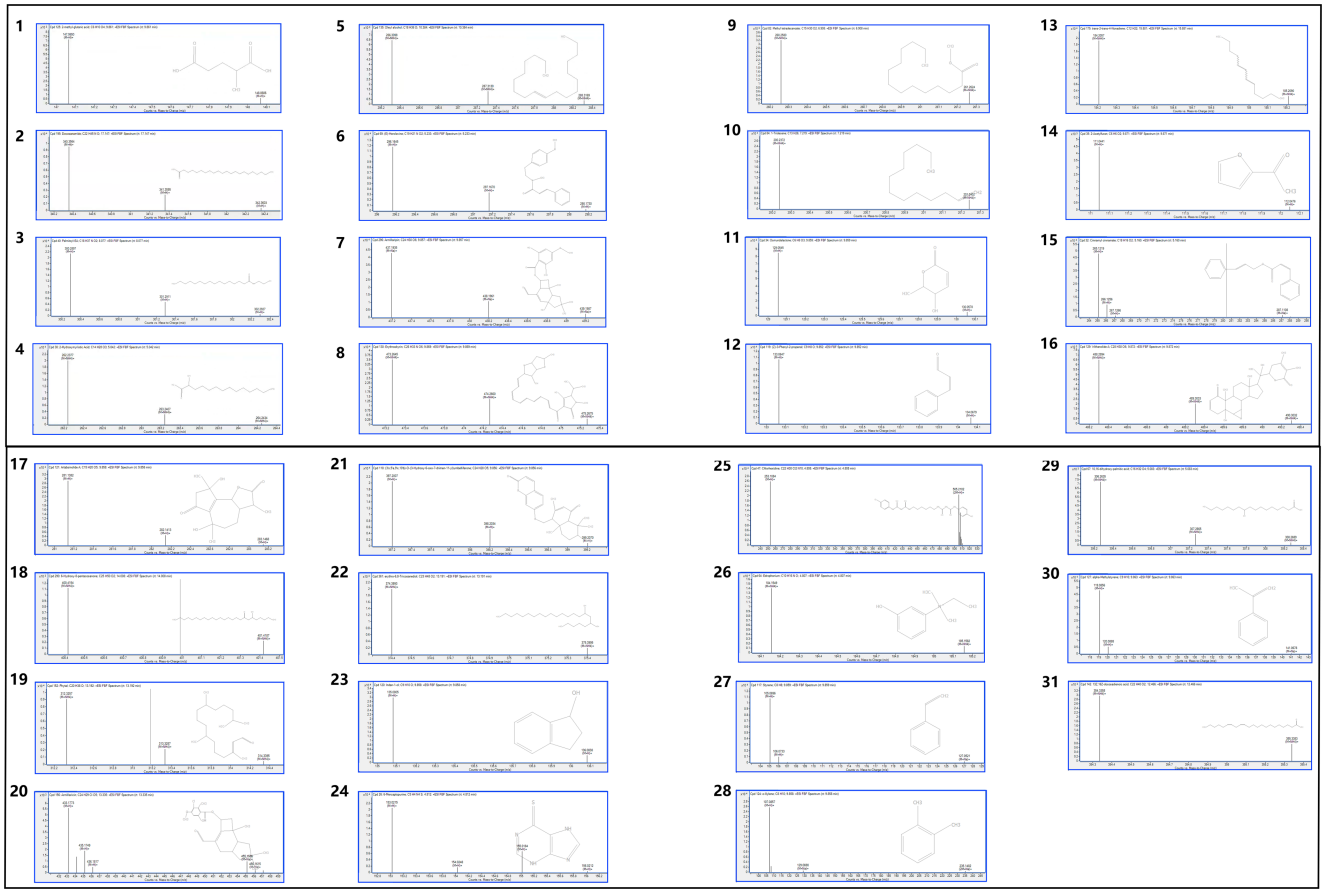
The mass spectrum of the differentiated compound.

Compounds with FDR < 0.05 and VIP > 1 were identified as differential metabolites, totaling 31 compounds, including: 2-Methylglutaric acid, Docosanamide, Palmitoylethanolamide, 2-Hydroxymyristic acid, Oleyl alcohol, (E)-Herclavine, Armillaripin, Erythroskyrin, Methyl tetradecanoate, 1-Tridecene, Osmundalactone, (Z)-Cinnamaldehyde, trans-2-trans-4-Nonadiene, 2-Acetylfuran, Cinnamyl cinnamate, Withanolide A, Artabsinolide A, 6-Hydroxy-8-pentacosanone, Phytal, Armillaricin, (3'x,5'a,9'x,10'b)-O-(3-Hydroxy-6-oxo-7-drimen-11-yl)umbelliferone, erythro-6,8-Tricosanediol, Indan-1-ol, 6-Mercaptopurine, Chlorhexidine, Edrophonium, Styrene, o-Xylene, 10,16-Dihydroxy-palmitic acid, alpha-Methylstyrene, and 13Z,16Z-docosadienoic acid. Among these, 9 compounds belong to Fatty Acyls, 5 to Benzene and substituted derivatives, 3 to Prenol lipids, 2 to Cinnamic acids and derivatives, 2 to Unsaturated hydrocarbons, and the rest are classified into Non-metal oxoanionic compounds, Carboximidic acids and derivatives, Organic phosphoric acids and derivatives, Furofurans, Pyrans, Cinnamaldehydes, Organooxygen compounds, Steroids and steroid derivatives, Lactones, Coumarins and derivatives, Indanes, Imidazopyrimidines, and Hydroxy acids and derivatives. The specific information is shown in Table 2. The mass spectrum of the differentiated compound is shown in Figure 5.

**Table 2:** The basic information of the 31 differential metabolites.

No.	Compound Name	Chemical Formula	VIP	m/z	RT	Ion Species	HMDB ID	Class	Sub Class
1	2-Methylglutaric acid	C6H10O4	1.407228369	147.0650	9.861286	[M + H]+	HMDB00422	Fatty Acyls	Fatty acids and conjugates
2	Docosanamide	C22H45NO	1.271730991	340.3564	17.147444	[M + H]+	HMDB00583	Fatty Acyls	Fatty amides
3	Palmitoylethanolamide	C18H37NO2	1.409097251	300.2897	8.077143	[M + H]+	HMDB02100	Carboximidic acids and derivatives	Carboximidic acids
4	2-Hydroxymyristic acid	C14H28O3	1.409379563	262.2377	5.0424294	[M + NH4]+	HMDB02261	Fatty Acyls	Fatty acids and conjugates
5	Oleyl alcohol	C18H36O	1.408221254	286.3098	10.384477	[M + NH4]+	HMDB29632	Fatty Acyls	Fatty alcohols
6	(E)-Herclavine	C19H21NO2	1.20291303	296.1645	5.23275	[M + H]+	HMDB30242	Cinnamic acids and derivatives	-
7	Armillaripin	C24H30O6	1.498546157	437.1935	9.857175	[M + Na]+	HMDB30404	Prenol lipids	Sesquiterpenoids
8	Erythroskyrin	C26H33NO6	1.474101452	473.2645	9.869257	[M + NH4]+	HMDB30464	Furofurans	-
9	Methyl tetradecanoate	C15H30O2	1.215490157	260.2580	6.9075556	[M + NH4]+	HMDB30469	Fatty Acyls	Fatty acid esters
10	1-Tridecene	C13H26	1.200519194	200.2372	7.2188125	[M + H]+	HMDB30930	Unsaturated hydrocarbons	Unsaturated aliphatic hydrocarbons
11	Osmundalactone	C6H8O3	1.406839683	129.0545	9.859429	[M + H]+	HMDB31303	Pyrans	Pyranones and derivatives
12	(Z)-Cinnamaldehyde	C9H8O	1.406503366	133.0647	9.852	[M + H]+	HMDB32072	Cinnamaldehydes	-
13	trans-2-trans-4-Nonadiene	C12H22	1.201197402	184.2057	15.880687	[M + NH4]+	HMDB32537	Unsaturated hydrocarbons	Olefins
14	2-Acetylfuran	C6H6O2	1.302724524	111.0441	9.870579	[M + H]+	HMDB33127	Organooxygen compounds	Carbonyl compounds
15	Cinnamyl cinnamate	C18H16O2	1.261071761	265.1219	5.159588	[M + H]+	HMDB33832	Cinnamic acids and derivatives	Cinnamic acid esters
16	Withanolide A	C28H38O6	1.408047691	488.2994	9.872429	[M + NH4]+	HMDB34415	Steroids and steroid derivatives	Steroid lactones
17	Artabsinolide A	C15H20O5	1.407195665	281.1382	9.857573	[M + H]+	HMDB35620	Lactones	Gamma butyrolactones
18	6-Hydroxy-8-pentacosanone	C25H50O2	1.210803489	400.4151	14.007502	[M + NH4]+	HMDB35629	Fatty Acyls	Fatty alcohols
19	Phytal	C20H38O	1.538749089	312.3257	13.192042	[M + NH4]+	HMDB35654	Prenol lipids	Diterpenoids
20	Armillaricin	C24H29ClO5	1.405564635	433.1773	13.334667	[M + H]+	HMDB38686	Prenol lipids	Sesquiterpenoids
21	(3'x,5’a,9'x,10'b)-O-(3-Hydroxy-6-oxo-7-drimen-11-yl) umbelliferone	C24H28O5	1.406902218	397.2007	9.855715	[M + H]+	HMDB39042	Coumarins and derivatives	-
22	erythro-6,8-Tricosanediol	C23H48O2	1.129635003	374.3993	13.191354	[M + NH4]+	HMDB41070	Fatty Acyls	Fatty alcohols
23	Indan-1-ol	C9H10O	1.407340829	135.0805	9.857572	[M + H]+	HMDB0059601	Indanes	-
24	6-Mercaptopurine	C5H4N4S	1.20230933	153.0215	4.81188	[M + H]+	HMDB0015167	Imidazopyrimidines	Purines and purine derivatives
25	Chlorhexidine	C22H30Cl2N10	1.25504509	253.1084	4.8078055	[M + H] + [2 M + H]+	HMDB0015016	Benzene and substituted derivatives	Halobenzenes
26	Edrophonium	C10H16NO	1.13010143	184.1549	4.8068333	[M + NH4]+	HMDB0015145	Benzene and substituted derivatives	Aniline and substituted anilines
27	Styrene	C8H8	1.406734828	105.0696	9.859428	[M + H]+	HMDB0034240	Benzene and substituted derivatives	Styrenes
28	o-Xylene	C8H10	1.407586764	107.0857	9.857573	[M + H]+	HMDB0059851	Benzene and substituted derivatives	Xylenes
29	10,16-dihydroxy-palmitic acid	C16H32O4	1.409150801	306.2635	5.0832863	[M + NH4]+	HMDB0037798	Fatty Acyls	Fatty acids and conjugates
30	alpha-Methylstyrene	C16H32O4	1.460029545	119.0865	9.862625	[M + H]+	HMDB0059899	Benzene and substituted derivatives	Phenylpropenes
31	13Z,16Z-docosadienoic acid	C22H40O2	1.215573431	354.3358	12.466	[M + NH4]+	HMDB0061714	Fatty Acyls	Fatty acids and conjugates

The Receiver Operating Characteristic (ROC) curve is a widely used method for evaluating the performance of binary classification models. It assesses the model’s classification capability by examining the relationship between sensitivity (true positive rate) and specificity (1 - false positive rate) across different thresholds. The diagonal line on the ROC curve represents random guessing, while curves closer to the top-left corner indicate stronger classification performance. Figure 3K indicates that in this study, ROC curve analysis revealed that 31 metabolites possessed an AUC value greater than 0.75. This indicates that these metabolites exhibit good accuracy and diagnostic efficacy in distinguishing between PD and non-PD cases.

**Figure 6:**
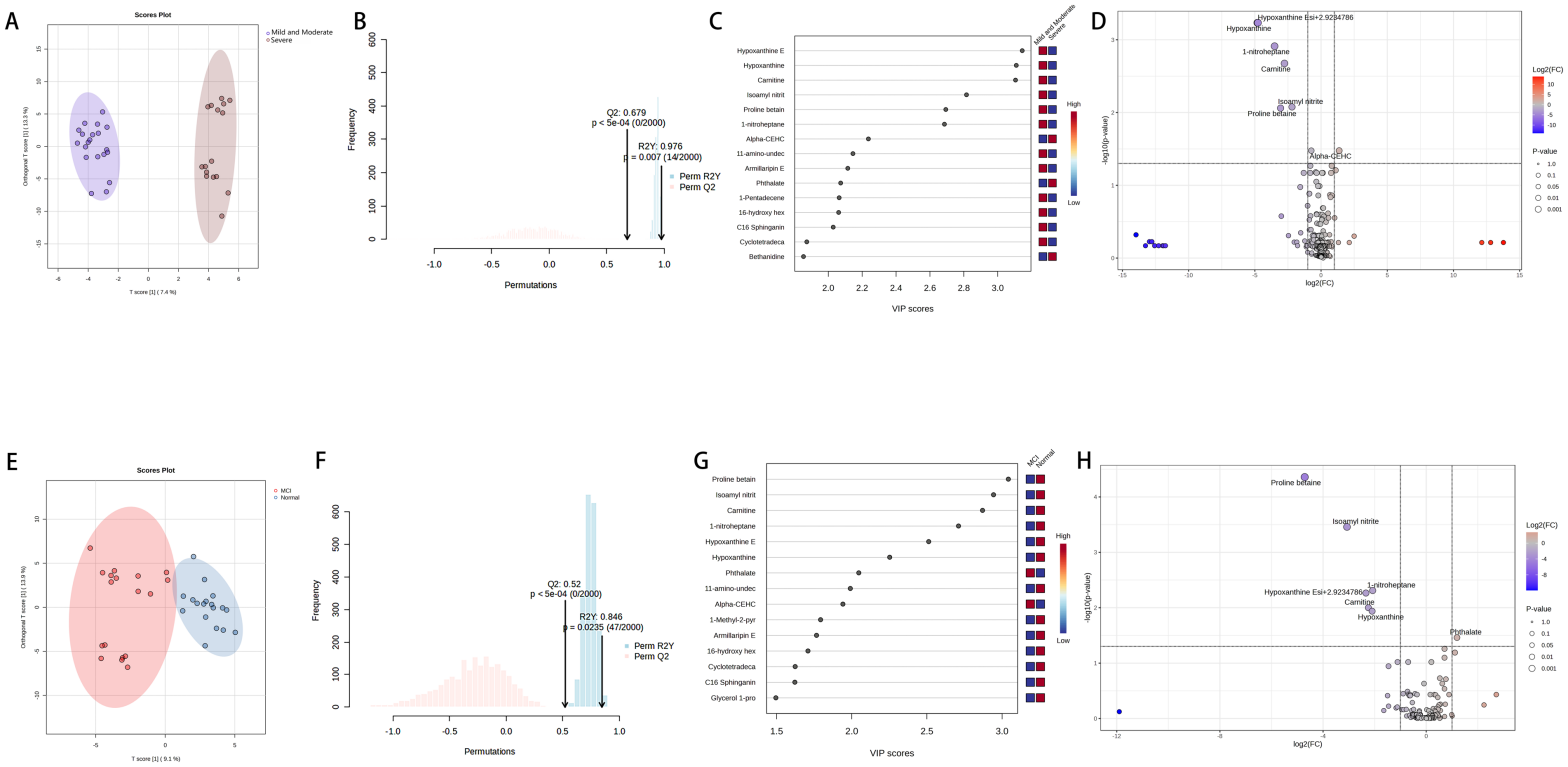
Panels **(A,B)** show the VIP, FC, and FDR visibility for metabolite comparisons between the mild-to-moderate and severe groups, and Panels **(C,D)** display the model results for these two groups, representing the metabolites in the PD group categorized according to the H-Y staging. Panels **(E,F)** show the VIP, FC, and FDR visibility for metabolite comparisons between the MCI and Normal groups, and Panels **(G,H)** display the model results for these two groups, representing the metabolites in the PD group categorized according to the MMSE scale.

As shown in Figures 6A,B, the Mild and Moderate group and the Severe group (20 vs. 16) within the PD samples are well-separated, with the model demonstrating strong fit (R2Y > 0.9) and predictive capability (Q2 > 0.6). Figures 6C,D illustrate the differential metabolites between the two groups, revealing downregulation of Hypoxanthine, Carnitine, Nitrite, and Proline betaine, alongside a notable upregulation of alpha-CEHC. As shown in panels E and F of Figure 6, the MCI and Normal groups in PD samples can be well separated, with the model’s R2Y > 0.8 and Q2 > 0.5, indicating good model fitting and predictive ability. Panels G and H display the differential metabolites between the MCI and Normal groups (18 vs. 18), with downregulation of proline betaine, isoamyl nitrite, hypoxanthine, 1-nitroheptane, and carnitine, while phthalate is upregulated.

#### 3.3.3 Random Forest

**Figure 7:**
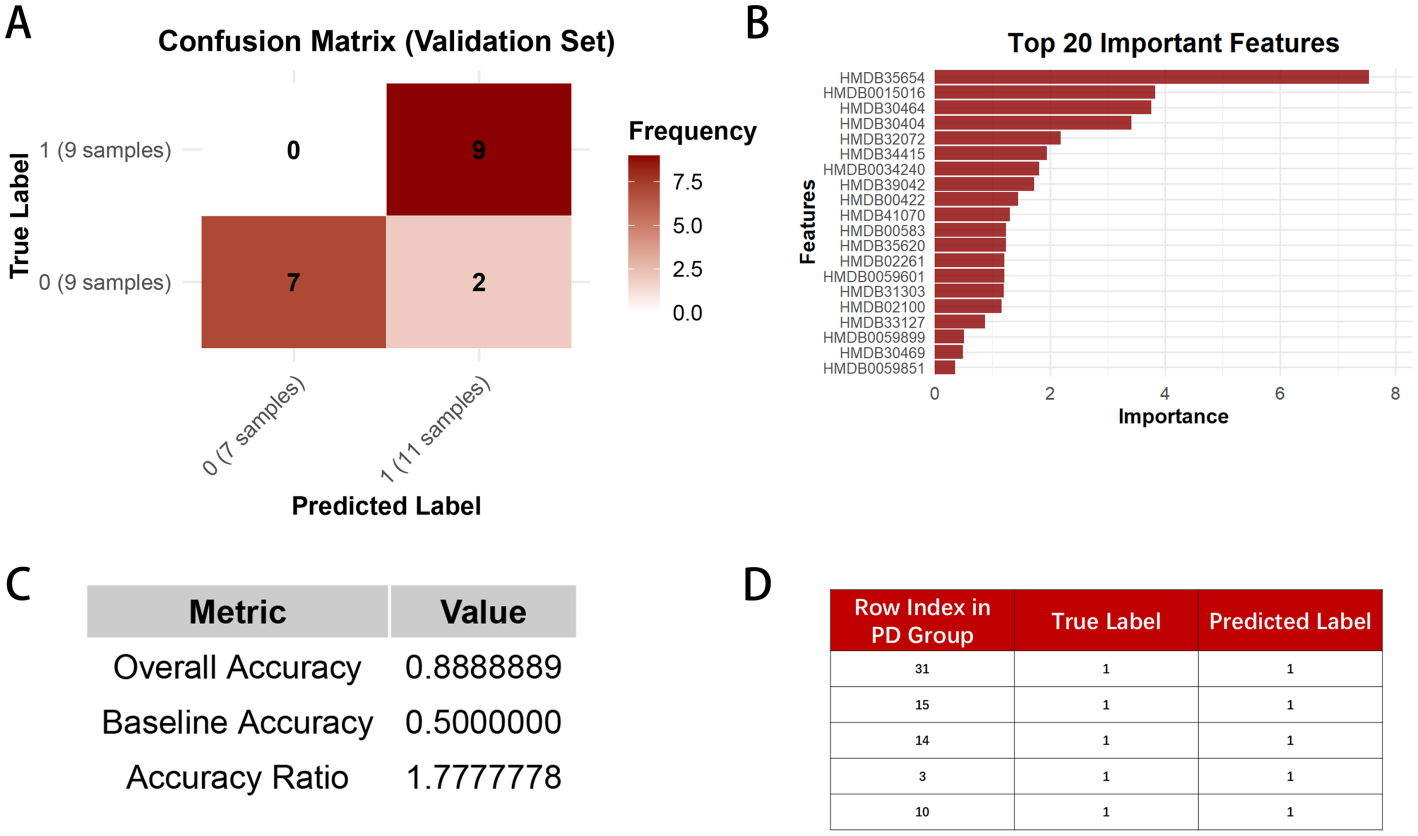
**(A)** represents the confusion matrix of the Random Forest model on the validation set, displaying the number of true positives, false positives, true negatives, and false negatives. In the matrix, 0 indicates the CON group, and 1 indicates the PD group. **(B)** shows the bar plot of feature importance in the Random Forest model. **(C)** presents the overall accuracy, baseline accuracy, and accuracy ratio of the model. **(D)** illustrates the prediction results of the five randomly selected independent validation set samples in the model.

In the Random Forest model constructed with 31 PD group samples and 31 CON group samples, 44 samples were allocated to the test set, and 18 samples were assigned to the validation set. Figure 7A shows that, in the validation set, our model correctly classified all 9 PD group samples. Among the 9 CON group samples, 7 were correctly classified, while 2 were misclassified as PD group. Figure 7C presents the model’s performance, revealing an accuracy of 88.9% on the validation set. This result appears considerably higher than the baseline accuracy of 50%, which may indicate the model’s potential utility, though further validation is required given the exploratory nature and limited sample size of this study. This further highlights the effectiveness and reliability of our Random Forest model. Figure 7B presents the top 20 important features, with the y-axis representing their HMDB IDs. The corresponding compound names can be found in Table 2. These features have a substantial contribution to the prediction results, suggesting that they are the primary drivers of the model’s predictions. Figure 7D shows the validation of the 5 randomly selected PD group samples, which were independently tested before the model was constructed. All samples were correctly predicted, demonstrating that our Random Forest model has high generalization capability and stability. Even with independent validation samples, the model can effectively identify key features and make accurate predictions. This high accuracy further enhances the model’s reliability in disease prediction.

## 4 Discussion

TCM has a long history in treating PD, and modern research in this field is also progressing rapidly. For example, studies have suggested that acupuncture holds significant promise as a therapeutic approach for PD, showing potential benefits in alleviating symptoms and improving patients’ quality of life (Zhuang and Wang, 2000; Zuo et al., 2022). This provides valuable insights into exploring TCM’s potential in diagnosing and treating PD. As a vital part of TCM, TC can provide a micro - level explanation of diseases through component research. Most studies by international scholars on TC have primarily focused on its association with halitosis (Roldán et al., 2005; Liu et al., 2006; Romano et al., 2020). Moreover, it is quite common internationally to use the complete removal of TC as one of the methods for treating halitosis (Kostka et al., 2008). Additionally, some international studies have explored the relationship between TC and systemic diseases beyond the oral and digestive systems (Kim et al., 2013; Mu et al., 2019; Hao et al., 2021; Xu et al., 2021), such as early rheumatoid arthritis (Kroese et al., 2021), pneumonia(Takeshita et al., 2010), lung cancer (Su et al., 2011), chronic renal failure (Gulsahi et al., 2014; Hao et al., 2019), coronary heart disease (Hao et al., 2019), perioperative conditions (Funahara et al., 2018), osteoporosis (Pereira et al., 2018), ischemic stroke (Huang et al., 2022), menstrual pain (Kim et al., 2017) and so on. However, most of these studies focus on the visual characteristics of TC, with limited investigation into its composition. Compared to the superficial visual features of TC, studying its composition offers deeper insights into disease mechanisms at the molecular level. Therefore, in clinical practice, when observing distinctive TC characteristics in outpatient cases, our team places greater emphasis on and interest in research focusing on its composition.

**Figure 8:**
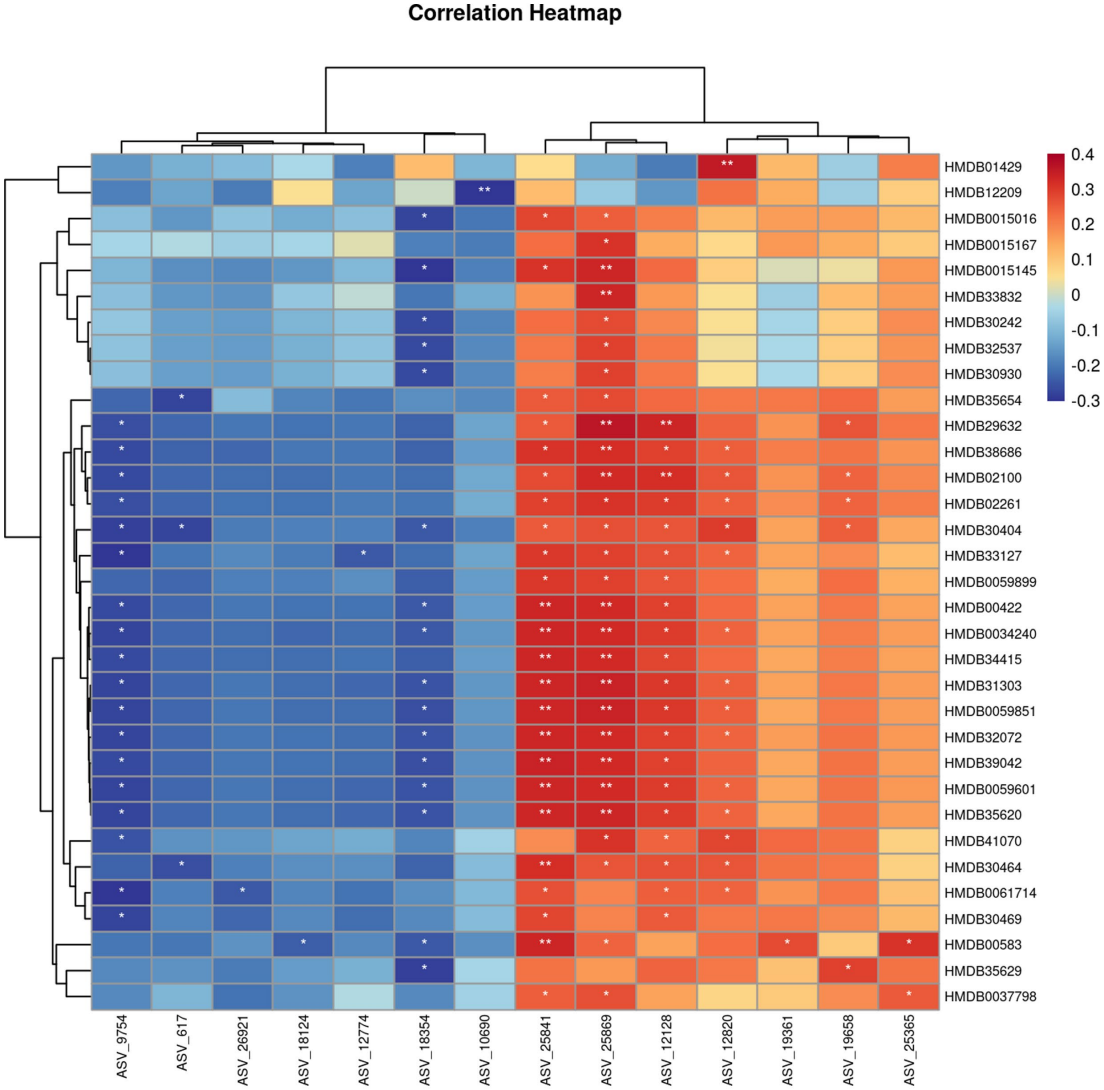
Spearman correlation analysis between metabolomics and microbiome. *: *p* < 0.05, **: *p* < 0.01.

Our experimental results suggested a significant difference in TCS between the PD and CON groups, prompting us to focus on molecular studies of TC composition to further explore the unique characteristics of PD patients. To our knowledge, no studies have yet investigated the microbial composition of TC samples from PD patients. However, studies (Fan et al., 2022; Hu et al., 2024) on fecal samples from PD patients have shown significant increases in the relative abundance of Proteobacteria, Firmicutes, and Actinobacteria at the phylum level, while Bacteroidetes exhibited a significant decrease. At the genus level, Streptococcaceae increased, and Veillonellaceae decreased, with LEfSe analysis showing significant enrichment of Bifidobacterium in PD samples. These findings align with the microbial alterations observed in TC samples from PD patients in our study. Compared to fecal samples, TC sampling is more easily accepted by patients and is also more convenient, clean, and safe for the operators. KEGG pathway analysis in our study suggested that metabolism-related pathways were notably enriched, which may reflect increased metabolic activity within the microbial community. This has sparked further interest in the metabolomics profile of the TC samples. So, we also opted for non-targeted metabolomics due to its high sensitivity, comprehensive nature, and ability to provide an intuitive reflection of biological states. The UPLC-Q/TOF-MS system was selected for its advantages, including high separation efficiency, exceptional mass resolution and sensitivity, rich structural information, and strong stability and reproducibility, making it highly suitable for analyzing a wide range of metabolites in complex biological samples. Given that most PD patients are elderly and often present with underlying conditions such as hypertension and hyperlipidemia, these factors are unavoidable in clinical sample collection. To mitigate their potential influence on our study, we selected family members accompanying patients for follow-up visits, matched by age group, as the CON group. This approach helped reduce the confounding effects of environmental and lifestyle factors. Statistical analysis showed no significant differences in the prevalence of hypertension and hyperlipidemia between the PD and CON groups, further enhancing the reliability of our research. At the same time, we combined image-based evaluation with metabolomics assessment to fully leverage the advantages of TC analysis. Our results suggested the robustness and reliability of our model. In our study, we identified 31 significant differential compounds. Some differential compounds, such as the upregulation of Docosanamide, originate from external contamination, while others, like the downregulation of Palmitoylethanolamide (PEA), are primarily derived from the body itself. This suggests that the disease state of Parkinson’s patients is influenced by both external environmental factors and internal factors. The utility of PEA has been widely studied, particularly in the context of neurodegenerative diseases. The mass spectrometry results of PEA are shown in Figures 4B,C. The Spearman correlation analysis between metabolomics and microbiome data is shown in Figure 8.

**Figure 9:**
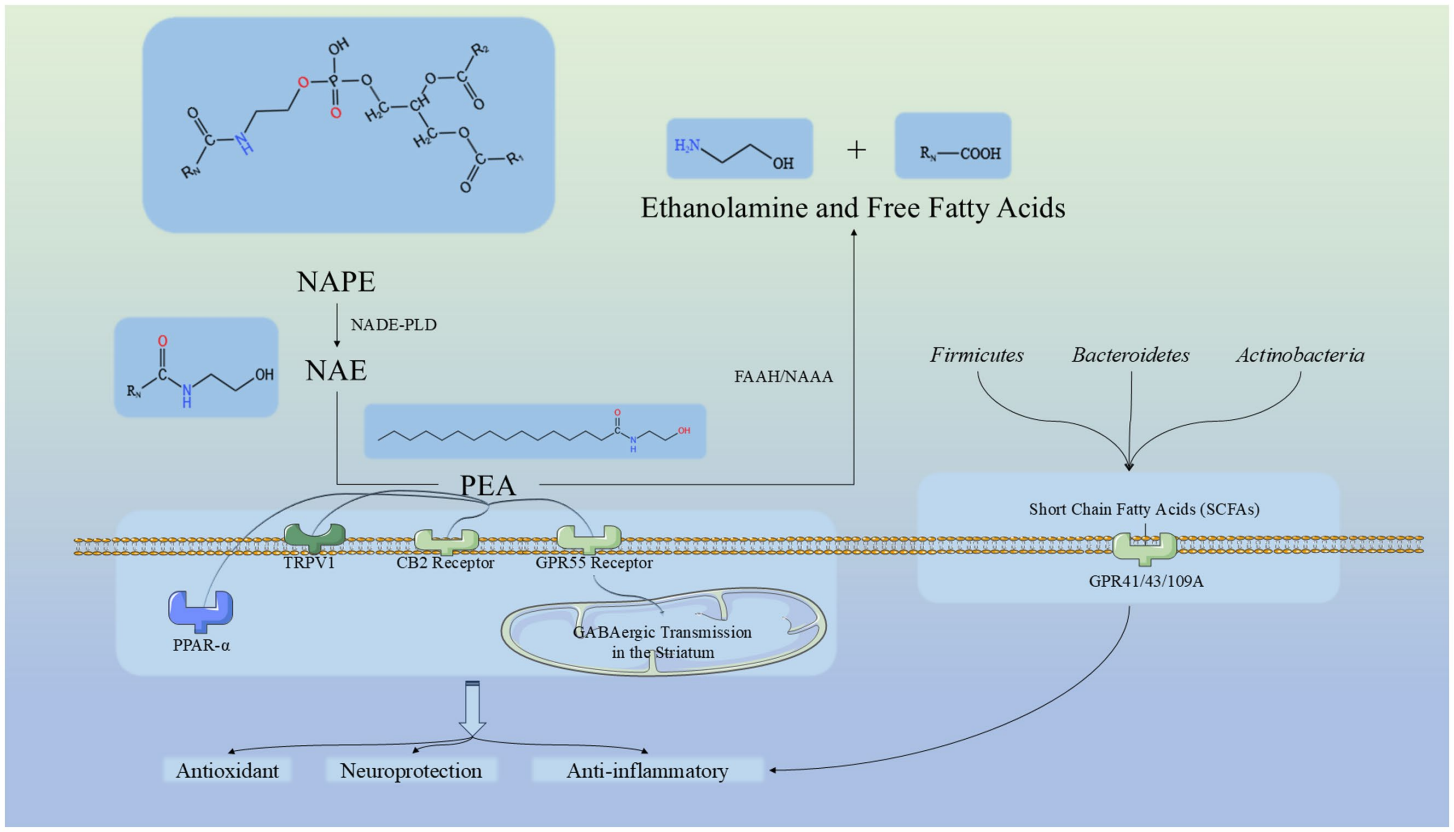
Pathway of PEA synthesis, metabolism, and mechanism, and its relationship with significantly enriched microbiota in this study. NAPE, N-acyl-phosphatidylethanolamine; NAPE-PLD, N-acyl-phosphatidylethanolamine-hydrolyzing phospholipase D; NAE, N-acylethanolamine; PEA, Palmitoylethanolamide; NAAA, NAE-hydrolyzing acid amidase; FAAH, Fatty acid amide hydrolase; TRPV1, Transient Receptor Potential Vanilloid type-1; PPAR-*α*, Peroxisome Proliferator-Activated Receptor Alpha, CB2 Receptor: Cannabinoid Receptor Type 2, GPR55 Receptor: G Protein-Coupled Receptor 55. GPR41/43/109A: G Protein-Coupled Receptor 41/42/109A.

The relationship between the oral-gut axis has been extensively studied. Building on this, research on the oral-gut-liver axis has also gained traction, leading to discoveries such as the connection between periodontitis and cardiovascular diseases (Koren et al., 2011; Acharya et al., 2017). This highlights the inseparable link between the oral cavity and the gut while offering promising avenues for exploring the oral-gut axis and its derivative pathways. For example, the oral-gut-brain axis. The gut microbiota plays a key role in communication between the brain and the gut (Aburto and Cryan, 2024). Increasing evidence suggests that impaired gut barrier function is associated with a wide range of central nervous system disorders, including neurodevelopmental, psychiatric, and neurological diseases. This has expanded the perspective of brain-related conditions to systemic diseases, with PD being one of the most extensively studied neurological conditions describing the relationship between the gut barrier and brain barrier (Aburto and Cryan, 2024). Studies have shown that one of the hallmarks of neurodegenerative diseases is alterations in the gut microbiota. PEA plays a role in several physiological processes directly related to maintaining gut barrier function, regulating inflammation and pain, and energy metabolism (Russo et al., 2018). PEA is an endogenous cannabinoid ethanolamide that acts as an “on-demand molecule” in the central nervous system, produced and released from neurons and glial cells. It is known for its analgesic, anti-inflammatory, and neuroprotective properties, achieved through the activation of various receptors and ion channels (Cordaro et al., 2018; Kiani et al., 2020; Cifelli et al., 2022). PEA belongs to the N-acylethanolamine (NAE) family, a group of bioactive lipids capable of modulating peripheral and central pathological processes (Russo et al., 2018). Neuroinflammation plays a crucial role in the pathogenesis of neurodegenerative diseases such as Alzheimer’s disease (AD) and PD (Siracusa et al., 2015; Crupi et al., 2018; Cordaro et al., 2020). PEA, with its potent neuroprotective and anti-inflammatory properties, is a key player in resolving neuroinflammation (Skaper et al., 2015; Cordaro et al., 2020; Petrosino and Schiano Moriello, 2020). Its primary target is the nuclear peroxisome proliferator-activated receptor-*α* (PPAR-α). Additionally, PEA can activate and regulate transient receptor potential vanilloid 1 (TRPV1) channels and indirectly activate cannabinoid receptors CB1 and CB2 (Esposito et al., 2012; Petrosino and Schiano Moriello, 2020; Cifelli et al., 2022). Through the CB2-mediated anti-inflammatory pathway, PEA modulates microglial polarization, reduces the release of pro-inflammatory cytokines, and enhances migration and phagocytic activity (Cifelli et al., 2022). By binding to GPR55 receptors, PEA enhances GABAergic transmission in the striatum and increases the postsynaptic synthesis of endogenous cannabinoid 2-AG, thereby regulating GABA release via presynaptic CB1Rs (Cifelli et al., 2022). This multifaceted mechanism endows PEA with exciting potential in treating PD and controlling its progression, as supported by various studies (Siracusa et al., 2015; Avagliano et al., 2016; Brotini et al., 2017; Crupi et al., 2018; Brotini, 2021; Palese et al., 2022). Moreover, PEA has shown promising effects in improving cognitive decline and mild cognitive impairment (MCI), particularly in the early stages of cognitive disorders (Calabrò et al., 2016; Colizzi et al., 2022; Landolfo et al., 2022). In our results, the PEA level in the PD group is significantly downregulated, making it an important factor distinguishing the PD group from the CON group. This further supports previous affirmations regarding the effects of PEA. Our findings indicate that the deficiency of PEA is a significant characteristic of PD patients and may even be a potential underlying cause of PD. This result is also evident at the level of TC, suggesting the potential for further research. It suggests that TC plays an important role in differentiating between individuals with or without PD. Additionally, since TC is a convenient and non-invasive sample type, it emphasizes its advantages and potential. Therefore, our study indicates that TC could serve as a promising sample for distinguishing PD, with PEA as a key biomarker for this differentiation. Given that PEA is endogenous, it is a more accurate reflection of the host’s condition. We have summarized the relevant routes of PEA based on literature, as shown in Figure 9 (Costa et al., 2008; Ueda et al., 2013; Aguilera et al., 2015; Musella et al., 2017; Lee et al., 2020; Mirzaei et al., 2021; Cifelli et al., 2022; Cong et al., 2022; Ikeda et al., 2022).

**Figure 10:**
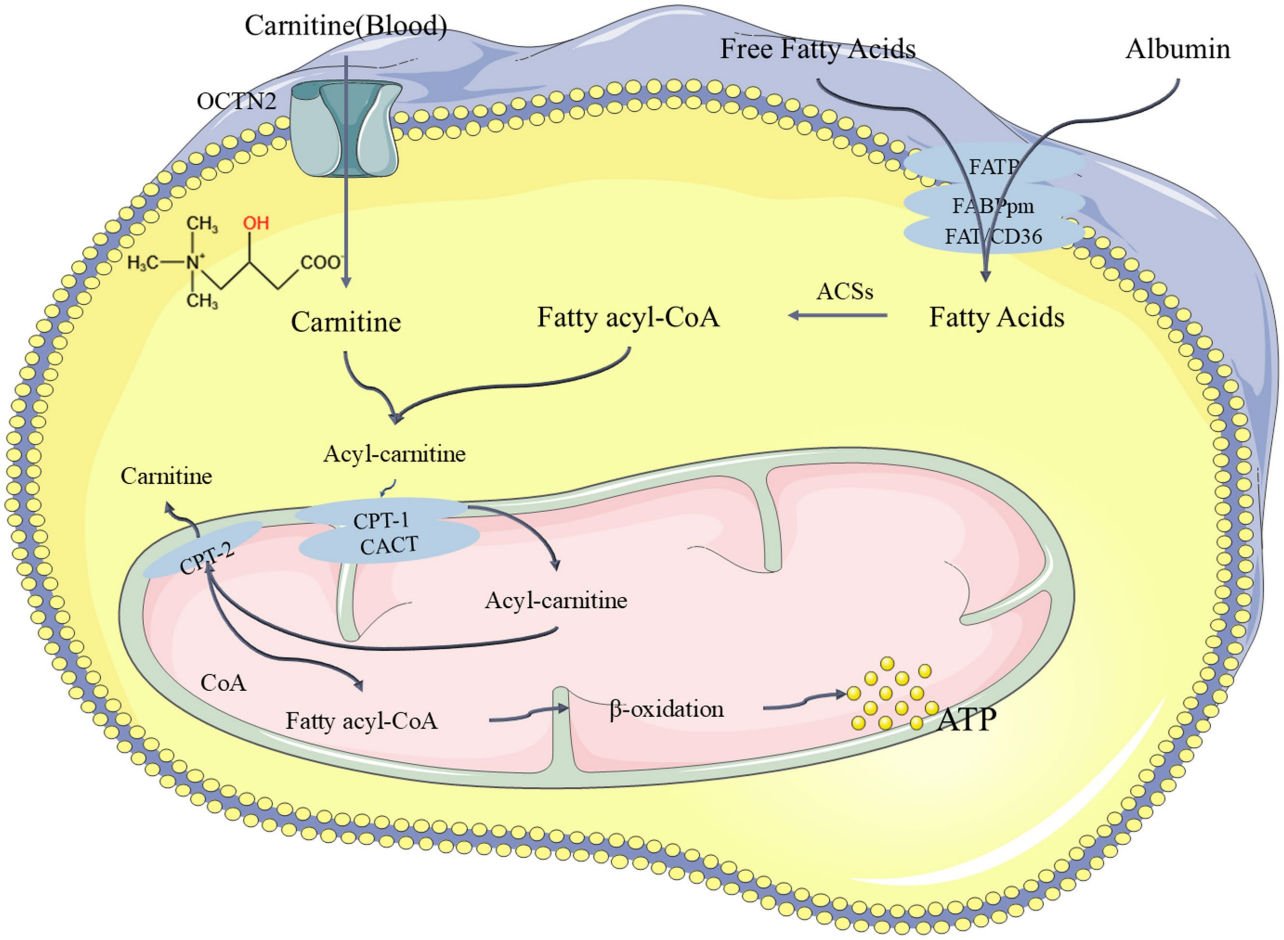
The pathway of carnitine undergoing *β*-oxidation in the body to release ATP and exert its function. FATP: Fatty Acid Transport Proteins, FAT/CD36: Fatty Acid Translocase, FABPpm: Plasma Membrane Fatty Acid Binding Proteins, ACSs: Acyl-CoA Synthases, CPT-1: Carnitine Palmitoyl Transferase 1, OCTN2: Organic Cation/Carnitine Transporter 2, CPT-2: Carnitine Palmitoyl Transferase 2, CACT: Carnitine-Acylcarnitine Translocase.

In our study, PEA appeared to play a significant role in differentiating between the PD and CON groups, but was not found to be significantly correlated with cognitive (based on MMSE scores) or disease progression (based on H-Y staging) groupings. This is because the difference in PEA levels between the CON and PD groups is substantial. In the CON group, the mass spectrometry peak value of PEA was relatively high, reaching hundreds of thousands, while in the PD group, the relative peak value was only 1. Therefore, PEA could be clearly separated between these two groups, where it serves an important role. However, when the relative peak value of PEA is uniformly 1 in the PD group across all samples, it cannot be used as a marker for cognitive or disease progression groupings based on H-Y staging or MMSE scores. Despite this, we identified an interesting metabolite in the cognitive and disease progression experiments: carnitine. Carnitine is a long-chain amino acid synthesized in the brain from essential amino acids lysine and methionine, which undergoes acetylation in the mitochondria to form acetyl-L-carnitine (Sergi et al., 2018). Many studies have focused on this compound, which has neuroprotective properties (Afshin-Majd et al., 2017; Gill et al., 2018). Some studies have also found that carnitine can prevent neuronal loss in Parkinson’s rat models and improve memory function (Singh et al., 2018). Recent studies have suggested a specific deficiency of free carnitine in early-stage Alzheimer’s disease (AD) in women. Patients with lower levels of free carnitine exhibit higher accumulation of *β*-amyloid (Aβ) and increased t-Tau levels in cerebrospinal fluid (CSF) (Bigio et al., 2025). In our results, carnitine levels were observed to be lower in both the severe H-Y stage group and the MCI group (based on MMSE scores), which aligns with the previously noted patterns. The mechanism of carnitine’s function in the body is shown in Figure 10 (Longo et al., 2016; McCann et al., 2021; Virmani and Cirulli, 2022). However, the relatively small sample size in the PD group limits the scope of these findings and warrants further investigation.

In addition, for the metabolomics data of the PD and CON groups, we also used the Random Forest method to build a robust and reliable model, which supported the predictive power of our biomarkers. This model suggested strong predictive performance under double validation, supporting the ability of our biomarkers to predict the disease and indicating a potential for the effectiveness of the biomarkers we identified.

The approach of combining microbiomics with untargeted metabolomics, using TC as a sample to identify biomarkers for neurodegenerative diseases, followed by validation through machine learning models, offers a promising new methodology. Compared to traditional sampling methods such as CSF, blood, urine, or feces, collecting TC is more convenient, less invasive, and more acceptable to patients. Moreover, it has greater potential for widespread adoption in community healthcare settings. If the sampling and processing procedures are thoroughly developed and standardized through research, patients could eventually collect samples themselves for basic testing. These findings suggest a potential foundation for future studies investigating the diagnostic and therapeutic role of TC in PD and other neurodegenerative diseases.

## 5 Conclusion

In conclusion, our study underscores the potential of TC samples as a non-invasive, cost-effective tool for diagnosing and monitoring PD. Metabolomics and microbiome analysis revealed differences between PD patients and controls, hinting at potential metabolic and microbial alterations relevant to PD. Key findings include reduced levels of palmitoylethanolamide (PEA) in PD patients, as well as a correlation between specific microbial phyla and differential metabolites. PEA deficiency may contribute to PD pathogenesis, but further studies are needed to fully elucidate its role. Moreover, the decreased carnitine levels observed in the severe H-Y stage and mild cognitive impairment (MCI) groups suggest a possible link to PD progression and cognitive decline. The application of machine learning techniques further supported the robustness of these findings, yielding an accuracy of 88.9%. This suggests that TC samples may hold potential as a tool for early PD diagnosis, a possibility warranting further investigation. These results suggest the clinical potential of combining metabolomics, microbiome analysis, and machine learning in advancing PD diagnostics and monitoring, which could lead to non-invasive, reliable tools for PD management. Most importantly, it offers new approaches and perspectives that could contribute to advancing PD research.

In summary, our findings suggest that conducting metabolomics and microbiomics studies using TC samples from PD patients provides an efficient means to uncover disease characteristics. However, the relatively small sample size and single-source outpatient data may limit the generalizability of our findings. A key aspect of our methodology was the implementation of strict inclusion criteria in our outpatient sampling. Our target population included PD patients committed to regular four-week follow-ups, and post-sample collection, any samples failing cell count standards were excluded. Consequently, only patients who successfully navigated all these rigorous screening and exclusion steps were included in the final study. We hope that through these stringent inclusion and exclusion criteria, our exploratory study, despite its small sample size, can provide a more robust data foundation. Our study supports the potential of TC samples as a non-invasive tool for PD diagnosis, but further validation in larger cohorts and multiple centers is required to confirm its clinical utility. Additionally, the current limitations in metabolite databases may have led to incomplete metabolite identification, highlighting the need for more comprehensive and updated reference databases to improve future research outcomes. The complexity of microbiome data, coupled with the limitations in functional inference, may result in the underrepresentation of certain microbial components. Future improvements could be achieved through more precise functional prediction models, enhancing the accuracy of microbiome analysis. Furthermore, variations in TC sampling methods across different research teams highlight the need for standardized protocols to ensure scientific rigor and consistency. Currently, we are actively strengthening our collaborations with neurologists and specialists. We plan to expand the scope of our research by conducting studies in multiple hospital environments. This strategy is expected to significantly increase our sample size and enhance the generalizability of our research findings. Future studies should focus on further validating the use of TC as a non-invasive sampling material, addressing standardization issues, and expanding its applicability in community healthcare settings.

## Data Availability

The original contributions presented in the study are publicly available. The raw microbiome data can be found at: https://www.ncbi.nlm.nih.gov, accession number: PRJNA1259750. The raw metabolomics data can be found at: https://www.ebi.ac.uk/metabolights/MTBLS12679.

## Footnotes

^1^ https://www.metaboanalyst.ca/

^2^ https://www.xsmartanalysis.com
