# Correction: Microbiome and metabolome integrated analysis: exploring potential diagnostic approaches for Parkinson's disease using tongue coating samples

**DOI:** 10.3389/fmicb.2025.1721341

**Published:** 2025-11-06

**Authors:** Runjuan Yang, Mengqi Jia, Ying Xu, Zhenghua Wu, Dongying Wu, Yaxing Gui

**Affiliations:** 1 Department of Clinical Pharmacy, Shanghai General Hospital, Shanghai Jiao Tong University School of Medicine, Shanghai, China; 2 Department of Neurology, Shanghai General Hospital, Shanghai Jiao Tong University School of Medicine, Shanghai, China; 3 School of Medicine, Shanghai University, Shanghai, China

**Keywords:** microbiomics, 16S rRNA, metabolomics, UPLC-Q/TOF-MS, Parkinson's disease, tongue coating

At their request, Guorong Fan has been removed as an author of this published article. His valuable contributions to the work are sincerely acknowledged in the updated Acknowledgments statement.

The correct author list is as follows:

Runjuan Yang, Mengqi Jia, Ying Xu, Zhenghua Wu, Dongying Wu, Yaxing Gui

The article citation, copyright statement, and correspondence section have been updated to reflect the removal of Guorong Fan from the author list of this article.

The corrected author contributions statement is as follows:

RY: Conceptualization, Data curation, Formal analysis, Investigation, Methodology, Writing – original draft. MJ: Conceptualization, Data curation, Formal analysis, Investigation, Methodology, Writing – review & editing. YX: Data curation, Formal analysis, Methodology, Writing – original draft. ZW: Data curation, Formal analysis, Methodology, Writing – review & editing. DW: Data curation, Formal analysis, Methodology, Writing – original draft. YG: Funding acquisition, Project administration, Resources, Supervision, Writing – review & editing.

